# Vaccine Hesitancy in China: A Qualitative Study of Stakeholders’ Perspectives

**DOI:** 10.3390/vaccines8040650

**Published:** 2020-11-03

**Authors:** Ronghui Yang, Bart Penders, Klasien Horstman

**Affiliations:** Department of Health, Ethics & Society, Care and Public Health Research Institute (CAPHRI), Maastricht University, P.O. Box 616, NL-6200 MD Maastricht, The Netherlands; b.penders@maastrichtuniversity.nl

**Keywords:** vaccine hesitancy, vaccine safety, organization of vaccination, incident response

## Abstract

A series of vaccine incidents have stimulated vaccine hesitance in China over the last decade. Many scholars have studied the institutional management of these incidents, but a qualitative study of stakeholders’ perspectives on vaccine hesitancy in China is missing. To address this lacuna, we conducted in-depth interviews and collected online data to explore diverse stakeholders’ narratives on vaccine hesitance. Our analysis shows the different perspectives of medical experts, journalists, parents, and self-defined vaccination victims on vaccination and vaccination hesitance. Medical experts generally consider vaccines, despite some flaws, as safe, and they consider most vaccine safety incidents to be related to coupling symptoms, not to vaccinations. Some parents agree with medical experts, but most do not trust vaccine safety and do not want to put their children at risk. Media professionals, online medical experts, and doctors who do not need to align with the political goal of maintaining a high vaccination rate are less positive about vaccination and consider vaccine hesitance a failure of expert–lay communication in China. Our analysis exhibits the tensions of medical expert and lay perspectives on vaccine hesitance, and suggests that vaccination experts ‘see like a state’, which is a finding consistent with other studies that have identified the over-politicization of expert–lay communication in Chinese public discourse. Chinese parents need space to express their concerns so that vaccination programs can attune to them.

## 1. Introduction

Vaccine hesitance, which is defined by the World Health Organisation (WHO) as a delay in the acceptance or refusal to vaccinate, despite the availability of vaccination services, has been reported in more than 90% of countries in the world [1]. Although China maintains a high vaccination rate of more than 90% for Category 1 vaccines (the Chinese vaccination program distinguishes between Category 1 and Category 2 vaccines. Category 1 vaccines are provided for free to all children until 14 years, and the use of these vaccines is considered a social duty. Category 1 vaccines include vaccines to prevent diseases, such as the hepatitis B vaccine, polio vaccine, and diphtheria–tetanus–pertussis (DTP) vaccine. Category 2 vaccines are optional vaccines that have to be paid for by the parents. The vaccines in this category include, for instance, vaccines to prevent human papillomavirus (HPV), mumps, rubella, pneumococcus, and rotavirus [2]), vaccine hesitance also exists in China. It has especially increased due to a series of ‘vaccination crises’ over the last decade [2,3]. In 2010, nearly 100 children suffered disability or death caused by adverse reactions to vaccinations in Shanxi province [4]. In 2013, eight babies died of adverse reactions after hepatitis vaccinations in southern China [5], and in 2016, many vaccines with unqualified cold storage entered the market in Shandong province, resulting in public anxiety [5]. In 2018, Changchun Changsheng Biotechnology Co., Ltd. produced invalid DTP vaccines, harming many children [6]. These incidents have all affected the public perception of vaccination safety in China. According to Liu [7], reports on a series of vaccination-induced cases of disability and death have caused vaccination hesitance among parents. Some parents have turned to alternative imported vaccines, which they assume to be safer and more effective. Others have refused vaccination altogether.

Considering these incidents, it is not surprising that most studies of vaccine hesitance in China have focused on the governance of vaccine safety by institutional and regulatory bodies [2]. Some Chinese scholars have assigned the responsibility for these safety incidents to the supervision system [6] or to the economic system of vaccine production [8]. Others have diagnosed vaccine hesitance as a problem of exaggerated media attention, arguing that the probability of an abnormal reaction to vaccination is very small—less than 1‰ [9,10]. Some scholars have also argued that imprecise media reporting stimulates parental risk perception of and doubts relating to vaccine safety, lowering the rate of Category 2 vaccination [11]. In addition, some have pointed to the role of professional medical conduct in stimulating vaccination hesitance and distrust [2]. Doctors exhibit little regard for pre-vaccination screening and post-vaccination observation and are not responsive to questions during vaccination, causing dissatisfaction with vaccination services among parents [12,13,14]. Meanwhile, healthcare professionals vaccinate without considering individual circumstances and demands, thereby sparking vaccine distrust [15]. Several studies have noted that unresponsive doctors destroy public trust [14,16], while others have argued that public distrust is the result of failed vaccination crisis management by both the state and experts [17,18]. However, according to Zhao et al. [19], it is a lack of public participation and information transparency in vaccine risk assessment, as well as suspicions about experts conspiring with the state, that have eroded expert credibility.

While these studies critically analyse the issue of vaccine hesitance in China, a qualitative study of how diverse stakeholders, including both experts, and lay individuals, consider vaccine hesitance in China is missing. To address this lacuna, we conducted a qualitative study to understand how diverse stakeholders give meaning to vaccine hesitance in China, with particular attention being paid to what they consider the main problem to be and which kinds of solutions they propose. In the next section, we describe our methods of data-gathering and analysis. After the presentation of the results of the analysis, we discuss and reflect on the findings and the difficulty of conducting a qualitative study on vaccine hesitance in China.

## 2. Materials and Methods

In this study, we adopted qualitative methods to gain insight into vaccine hesitancy in China. According to Small and Saunder et al., contrary to the sample-based logic of quantitative surveys, qualitative research allows for rich, high-quality data that do justice to diverse individual perspectives and personal accounts. Therefore, qualitative research has strengths in terms of the quality of data that compensate for the lower number of participants [20,21]. According to Morse, on the basis of data saturation, comprehensive theories can be developed [22]. In line with this, to do justice to a broad variety of narratives and perspectives, the first author conducted semi-open interviews with diverse stakeholders, including two Center for Disease Control (CDC) experts, six vaccination doctors in hospitals [23], two online medical experts (online medical experts refer to the medical experts who provide medical consultation via the Mobile Health Application), a consultant in vaccine manufacturing, four parents, and two journalists. The interviews took place in the cities of Shanghai, Wuhan, and Xinyang from January to February 2019. Engaging parents in interviews proved challenging, which was likely due to political sensitivities. To do justice to lay perspectives, we also analysed detailed online narratives offered by three self-defined ‘victims’ of vaccination. The interviewees were purposefully selected: Participants were approached because of their knowledge of and experiences with vaccination.

Performing a qualitative study in China is quite complicated methodologically. In preparation for this study, the first author first unsuccessfully initiated formal interviews with stakeholders regarding vaccination attitudes in China. The sensitive political environment makes it impossible to get access to potential participants via standard formal procedures [24]. CDC experts were cautious about participating in field investigations for fear of negative reports, damaging the governmental image, and the consequential administrative accountability. Clinicians in hospitals who were busy with the task of vaccination and Chinese citizens both generally distrust interviews conducted by strangers, and were reluctant to openly express personal opinions about politics and policies, as such individuals fear punishment by their leaders and social reprisal if they express ideas that deviate from official policies. To deal with these difficulties, we thus followed informal strategies to recruit participants, such as acquaintance mediation, befriending, and gift-giving. This allowed us to establish a private, trusting relationship with potential participants before collecting data. Meanwhile, the snowballing selection of participants was employed to include participants’ colleagues.

When inviting people to participate, the first author underlined that the aim of the study was academic, not political or commercial, as state-salaried experts are not encouraged to involve themselves in interviews without state authorization. Prior to the interviews, we obtained the oral informed consent of research participants after sharing with them the research goals, methods, funding sources, expected outcomes, anticipated impacts, and rights and responsibilities of the participants, as well as establishing their anonymity. To facilitate interviewees telling their stories, the interview location selection was kept flexible, including tea bars, cafés, hospitals, and interviewees’ homes. During the interviews, the interviewees did not allow the interviews to be recorded, as they were worried about their privacy. The first author thus took intensive notes during and after the interviews, and after discussing the notes with the whole team, relevant sections were translated into English.

Restrained by states, there is less public discussion about vaccine hesitance on Chinese mainstream social media platforms, such as Baidu, WeChat, and Zhihu communities. Therefore, we collected stories of three self-defined victims that were shared on the online Chinese forums of the Tianya community and Sina Weibo. The stories of self-defined vaccination victims are interesting, as these accounts are less self-censored. On these online forums, one can speak relatively freely, as local governments believe that in an era of information explosion, online opinions with low click rates will not cause negative social effects, rendering governmental intervention unnecessary [25]. These online comments tend to focus on the personal storytelling of vaccination-induced events, criticism of the state performance, and policy suggestions regarding the National Immunisation Programme (NIP). The lengths of these comments varied from one sentence up to 2500 words. These comments were posted during the period of vaccine incidents between 2013 and 2018. We selected the stories of three self-claimed vaccination victims based on the time of the post and the richness of the content. To protect the privacy and anonymity of the research participants, we assigned them codes: ‘ID X’ (see Table 1 and Table 2). (To ensure the quality of the data, the accuracy and consistency of interview notes translated from Chinese were verified by Liang Yu, Associate Professor of Tianjin University, China).

To analyse the transcripts of the interviews and the online stories, we conducted a thematic analysis. Important themes that we identified were the safety of vaccines, the practical organization of vaccination, and responses to vaccine incidents. We identified more detailed sub-themes via deductive, iterative coding of the data: Signed informed consent; combined and single vaccinations; antibody testing; vaccine incident investigation(s); information disclosure; and the diagnoses of side effects. Subsequently, exemplary data extracts were selected from the key sub-themes for inclusion as quotations [26].

## 3. Results

The analysis of the perspectives and experiences of medical experts, clinicians, parents, and journalists on vaccination is presented in three sections. First, we present the perspectives on vaccine safety. Second, we explore perspectives on the practical organization of vaccination. Finally, we deal with the different perspectives on vaccination crisis responses.

### 3.1. Perspectives on Vaccine Safety

Notably, notwithstanding various vaccine safety incidents over the last few years, a CDC expert, two clinicians, and two parents explained that there was no structural and fundamental safety issue with domestic vaccines. They claimed that the efficacy of vaccines had been undermined by illegal corporate production, but that the safety of vaccines themselves was qualified (ID 1, 15,16); strict electronic monitoring and a risk response system had been established by the state, so unqualified vaccines are now rapidly controlled and vaccine safety incidents no longer occur.

The efficacy of vaccines produced by Changsheng decreased, but the safety of vaccines meets the standards, which means that it will not threaten citizens’ lives. (ID 1)

During the Changsheng vaccine crisis, regulatory bodies uncovered falsifying data for the rabies vaccine and then controlled these problematic vaccines promptly. No faulty vaccine was consumed by citizens. (ID 6)

Additionally, re-vaccination campaigns were conducted by the state to ensure that antibodies were produced. (ID 3)

Along similar lines, the interviewed CDC expert pointed to bad reports in the media and unclear personal cognition of the hazards of epidemics and the benefits of vaccination, both of which raise the risk perceptions of parents:

Media reports included these casualties as a result of unsafe vaccine uptake and stimulated parental anxiety via rapid information dissemination online. (ID 2)

‘People cannot give up eating for fear of choking’. They should not hesitate to vaccinate. An effective crisis response should be conducted to mitigate public disquiet. (ID 5)

Nonetheless, online medical experts established the opposite. They indicated that these less effective vaccines not only fail to prevent viral infections, but also threaten children’s lives (ID 10). According to them, most of the vaccines on the market are unqualified, which they attributed to illegal corporate production, unqualified cold storage, regulation deficits, and state–business collusion. One public expert acknowledged that Changsheng employed large tanks to produce vaccines instead of state-authorized canisters in an effort to maximize profits, generating great potential risks in vaccine safety (ID 11).

People may relax their vigilance and exercise less, as they believe that the antibodies produced after vaccination lead to decreased immunity, especially the invalid rabies vaccine, threatening people’s lives. (ID 10)

Meanwhile, changes in the contents of culture containers produce new, unknown substances which current technologies fail to assess, increasing risks in vaccine safety. (ID 11)

In a similar way, journalists and online medical experts noted that doctors are known to pile vaccines on easily accessible tables without cold storage at the county level to finish vaccination assignments quickly, instead of caring about public health (ID 10, 12, 13):

I saw the doctor throw vaccines on the table without cold storage and pile them randomly, which made me anxious about vaccine safety. During the vaccination, I kept asking: ‘Is this kind of vaccine—please don’t take it wrong—is it safe without cold storage?’ (ID 13)

One parent argued that the regulatory bodies had adopted a random regulation model where only 5% of vaccines were selected for examination, resulting in large numbers of unqualified vaccines failing to be screened (ID 17). State–business collusion had also induced regulation deficits, as well as unsafe vaccines, which one parent believed should be reported (ID 18). Vaccine manufacturers are keen to maintain private relationships with local officials; consequently, illegal corporate production occurs and the regulatory capacity is eroded:

The vaccine regulatory system is strict in China, but why were falsified vaccine quality reports from the Chansheng company failed to be screened by the state? State–business collusion and bribery should be to blame. (ID 18)

An online medical expert indicated that CDC experts in public institutions are not involved in conversations related to negative information about vaccines, as they themselves undertake vaccination tasks, maintaining a high vaccination coverage and scientific knowledge popularization:

CDC experts are assigned with the responsibilities of immunization, epidemic-preventing and science popularisation regarding public health. When vaccination incidents occur, CDC experts are obliged to conduct crisis management and reassure the public. Additionally, CDC experts are able to release negative information concerning vaccination with the state permission, otherwise they will be accountable for damaging the reputation of the state (ID 10).

To secure vaccine safety, both the online medical experts and the parents suggested reducing the economic incentives of manufacturers, expanding samples for inspection, and restraining power-renting (ID 10, 11, 15):

More vaccines should be freely provided by the CDC, more than 5% of vaccines should be selected for examination, and a strict administrative accountability mechanism should be conducted to impose punishments on manufacturers and officials. (ID 10)

In summary, CDC experts and clinicians claimed that vaccines were safe in China, and that the strict regulation system plays a critical role in this. According to them, high personal risk perception should be attributed to negative reports by the media, and the narratives of online medical experts and the media are fundamentally opposed to each other: Online medical experts and journalists claimed that the invalid vaccines produced by manufacturers are fatal, putting citizens at risk. The parents, meanwhile, perceived deficits in the regulation system and called for reform.

### 3.2. Practical Organization of Vaccination

Alongside the vaccine safety issue, participants also presented their ideas on the practical organization of vaccination, which involves three topics: Pre-vaccination informed consent forms; combined vaccinations; and post-vaccination antibody tests.

#### 3.2.1. Signing Pre-Vaccination Informed Consent Forms

CDC experts argued that signing pre-vaccination informed consent forms facilitates the organization of immunization programs, as it defines doctor–patient responsibility and reduces medical tensions, stimulating public understanding of vaccination expertise (ID 1, 2).

More than 80% of doctor-patient tensions result from unclear responsibility-defining. Informed consent signing clearly allocates blame and mitigates tensions. (ID 2)

In similar ways, one parent considered disease-preventable vaccines, the types and cost of vaccines, contraindications (conditions in a recipient that increase the risk for a serious adverse reaction), adverse reactions, and precautions to all be clearly elaborated in informed consent forms (ID 15). Clinicians argued that informed consent signing normalizes vaccination procedures, increases doctors’ communication awareness, promotes information transparency, and stimulates public understanding of vaccination expertise (ID 4, 6, 7).

Doctors will cautiously screen children’s health situations before vaccination, interact with the public about vaccination precautions, and record vaccination procedures. (ID 4)

Nonetheless, informed consent signing was criticized by the online experts and three parents, who considered it a strategy employed to deal with medical tensions and to exempt doctors from vaccination incidents. As CDC undertakes immunization tasks, they are undoubtedly confronted with doctor–patient tensions caused by vaccination side effects while maintaining a high vaccination coverage (ID 10). On the other hand, doctors claim ‘no signing, no vaccination’, forcing parents to give their informed consent before vaccination, which some interviewees believed ignores public benefits and is unjust (ID 16, 18). They also argued that, in practice, doctors are over-dependent on informed consent, stressing that parents read cautionary statements on the informed consent form without interacting with parents themselves (ID 19).

Because informed consent stresses parental duties but neglects to define doctors’ responsibilities, I failed to hold doctors accountable after a side effect harmed my child. (ID 10)

Doctors ask parents to read vaccination information on the informed consent form instead of informing them of precautions. This way, they do not have to interact with parents. (ID 19)

Parents suggested the following strategies to deal with public doubts on informed consent signing, including listing all of the possible abnormal reactions, defining remedies, and improving doctors’ responsiveness (ID 19, 20):

All side reactions and remedies should be listed clearly, including the training that doctors have done to improve their social responsibility. (ID 20)

Most clinicians, CDC experts, and vaccination doctors, as well as two parents, noted that informed consent facilitates the defining of doctor–patient responsibility, reducing medical tensions, stimulating public education, and improving doctors’ responsiveness, while online medical experts criticized informed consent for exempting doctors from responsibility for incidents. Most of the parents also pointed out the injustice of compulsory informed consent and less interaction with doctors caused by over-dependence on informed consent.

#### 3.2.2. Single or Combined Shots

Concerning vaccine choice, one CDC expert recommended the separation of Category 1 and Category 2 vaccinations to reduce their mutual influence and to better identify which vaccines cause side effects.

Combined vaccination may interfere with producing different antibodies, increasing adverse effects. On the contrary, a single shot is favourable to defining the responsibilities between CDC and the medical institution. (ID 2)

The CDC expert also pointed to the commercial nature of combined vaccines. Manufacturers and doctors exaggeratedly advertise their health benefits and vilify separate vaccinations to encourage combined shots (ID 1). Meanwhile, despite their high prices, some parents noted that combined vaccines are as effective as free single vaccines in terms of disease prevention (ID 16, 18).

Doctors recommend combined shots, as children benefit from [them]. These commercial combined shots are around 4000¥, and I feel pressure to pay. I prefer the free single shot. ‘Free’ does not mean bad but is the best for my child. (ID 16)

One parent doubted the immature production technology and unknown risks of combined vaccines, as the practice has not been popularized nationwide by the state (ID 20). However, clinicians argued that combined shots were safe, were performed globally, and increased the efficiency of vaccination, as they involve fewer hospital visits, reducing the pain of injection and vaccination-induced side effects (ID 3, 7, 12).

Diphtheria, Tetanus, acellular Pertussis, Inactivated Polio Vaccine (DTaP-IPV), adopting advanced technologies to quantitatively ration antigenic components, ensures solubility, physical compatibility and stability. (ID 3)

Four doses of DTaP-IPV injection with children instead of 12 doses of single shots greatly reduces the number of side effects and saves time in visiting the hospital. (ID 11)

As such, clinicians and journalists suggested that the state should provide policy support for combined vaccine innovation and clinical research and improve the combined vaccine industry infrastructure (ID 5, 6, 12, 13).

No manufacturers in China so far can produce the DTP vaccine, polio vaccine or DTaP-IPV vaccine. Therefore, the state should increase investment in technology innovation and promote the cooperation of manufacturers and research institutes to overcome technical bottlenecks and stimulate industrial upgrading. (ID 6)

Many clinicians and parents essentially took this view, considering combined shots to be safe and convenient. CDC experts, on the other hand, proposed single shots to better define the responsibilities of the CDC and the hospital for vaccination-induced side effects, and noted the commercial purposes of combined shots. Similarly, a few parents were concerned about the potential risks of combined vaccines.

#### 3.2.3. Post-Vaccination Antibody Tests

CDC experts and clinicians argued that not everyone needed a post-vaccination antibody test. As the NIP adopts the ‘herd immunity’ strategy, this ensures a form of indirect protection from disease that occurs when most of a population has become immune to an infection, thereby providing a measure of protection for individuals without antibodies produced after immunization (ID 1, 2, 5, 8).

Herd immunity greatly increases the efficiency and reduces the costs of vaccination programmes. As long as everyone can be vaccinated in a timely manner, individuals vulnerable to infection without antibodies will be protected, and the disease will inevitably be eliminated (ID 5).

Additionally, one CDC expert noted that the antibody reagents were sometimes insensitive, causing inaccurate antibody tests. For instance, fewer antibodies are generated in blood circulation, which cannot be detected by current technology, but this does not entail that children do not have the antibodies (ID 2). Instead, the observation method could be a method employed to assess antibody presence (ID 16).

Bacteria generated by uptake of live bacterial vaccines need to grow and multiply in individual bodies to activate the immune system, and there will be a skin reaction within 2–3 months, which indicates induced immunity. (ID 16)

However, journalists and online medical experts suggested that antibody tests need to be designed through technology, as personal experience is less credible and the success rate of vaccination is not perfect. Children should be tested for reassurance, they said, rather than blindly re-vaccinated (ID 10–13).

During the Changsheng invalid vaccine incident in 2018, several local governments conducted re-vaccination campaigns, but they only increased the chances of side effects. I suggest testing antibodies first. (ID 12).

As such, parents suggested two schemes to test for antibodies via technology. One group of parents suggested visiting antibody test agencies in Hong Kong, considering its good antibody test infrastructure and convenient transportation (ID 17). Another group of parents called for developing antibody test infrastructure on the mainland (ID 14, 15).

I took planes to go Hong Kong with my child and finish antibody testing on the same day. Then I received the report quickly. (ID 17)

While the cost of antibody tests in Hong Kong is expensive, antibody tests relate to public health. The state should build laboratories to provide test services. (ID 15)

In summary, CDC experts argued for no antibody testing, considering its insensitivity and their adoption of the herd immunity strategy. A few parents suggested assessing antibodies through observation. Journalists and online medical experts opposed both of these suggestions and argued for antibody tests for reassurance. Consistent with this advice, many parents proposed conducting antibody tests in Hong Kong and called for developing antibody test infrastructure in mainland China as well.

### 3.3. Incident Response

In this section, Chinese stakeholder arguments on adverse vaccination-induced events are presented, involving vaccine incident investigations, information transparency, and diagnosing adverse effects.

#### 3.3.1. Vaccine Incident Investigations

When adverse vaccination-induced events occur, CDC experts are authorized to conduct risk assessments to ascertain causality and responsibility, but perspectives on this procedure also differ. According to the interviewed CDC experts, to ensure justice during the procedures of data collection and risk assessment, strict norms for data collection (ID 4, 5) and entry qualifications of experts for risk assessment are necessary (ID 1, 2).

There is at least one clinical expert participating in data collection to obtain detailed information about children and doctors. (ID 4)

Experts eligible for risk assessment should have professional knowledge and extensive clinical experience and should be selected randomly from expert databases. Those who keep relations with victims and vaccinators should be excluded. (ID 2)

However, these standards of risk assessment were questioned by many parents. To avoid conflicts of interest, the central state has developed polices that forbid vaccination experts to become engaged in risk assessments related to vaccination incidents, but in practice, local CDC experts who conduct vaccination are often involved in such risk assessment and steer decision-making (ID 14, 19). Meanwhile, there is a lack of specific technical standards for risk assessment, leading to different diagnoses for incidents in different regions (ID 18).

The CDC is not only the athlete but also the referee in the risk assessment, as risk assessment experts keep close ties with local CDCs and prioritise their interests (ID 19).

Experts make subjective decisions based on partial medical records and personal experience. (ID 18)

One clinician countered that expert risk assessment was just, as there is no absolutely reliable technology for ascertaining causality; the best technicians make the best diagnoses for vaccine incidents (ID 6). To mitigate these disputes between experts and the lay public, the latter should be encouraged to participate in risk assessments to judge, discuss, debate, and compromise (ID 10). Furthermore, considering that lay experience might be marginalized in risk assessment, as experts take the experience of the public for granted as a constraint, one journalist and online medical experts argued that the lay public could delegate expert agents and third-party institutions to assess risk, in order to maintain their benefits and values (ID 10–13).

Parents know children’s health situations and witness vaccination procedures. Their involvement in risk assessment will contribute to better judgement, negotiation, information transparency and trust. (ID 11)

In summary, CDC experts and clinicians valued the risk assessment procedure just as they valued strict norms for data collection and entry qualifications of risk assessment experts, while many parents questioned the procedure’s justice and pointed to CDC’s steering risk assessments and the subjective diagnoses of experts. Journalists and online medical experts proposed public engagement to mitigate this distrust.

#### 3.3.2. Information Disclosure

Information disclosure relates to scientific risk assessment and blame allocation [2]. Interviewed CDC experts indicated that the state had established specific norms for information transparency and regularly evaluated the extent of information disclosure at the local level (ID 1, 2).

Laws have defined specific standards for information disclosure. To stimulate transparency, the state conducts rigorous annual evaluations at the local level. (ID 2)

Different from the routine period, clinicians and one parent argued for the rationality of maintaining positive, limited information disclosure during crises to mitigate public panic, as they believed that absolute transparency would stimulate distrust and increase supervision difficulty (ID 7–9, 16).

During the hepatitis B vaccine [incident] in 2013, the government kept the investigation process transparent, which in turn fed public fear. (ID 7)

However, journalists and online medical experts argued that accountability required information transparency. Limited information disclosure spurs local officials into suppressing information dissemination and encourages experts to privatize data, resulting in less media concern and a general failure of public accountability (ID 10–14, 17, 18). As a response, they proposed solutions such as traceability systems and live streaming for monitoring vaccine incident investigations (ID 6, 10).

Public officials will not release unfavourable information, aiming to shirk responsibility. Experts will claim ‘coupling symptoms’ while keeping evaluation procedures, techniques and standards opaque. (ID 17)

The traceability mechanism records details of crisis management to keep information relevant to victims, vaccination, diagnosis, data collection and risk assessment. Live streaming can be adopted to ensure the procedure’s transparency. (ID10)

In summary, CDC experts and clinicians argued, on the one hand, that established standards were capable of keeping information transparent at the local level. They also stressed limited information disclosure during crises to reassure the public. Journalists and online medical experts, on the other hand, countered that accountability requires information transparency and that information asymmetry encourages buck-passing. As a response, they called for traceability systems and live streaming to maintain procedure transparency.

#### 3.3.3. Diagnosing Adverse Effects: ‘Coupling’ or ‘Malpractice’

One of the themes in the interviews was diagnosing adverse effects. The interviewed CDC experts argued that most of the abnormal reactions to vaccination were coupling symptoms related to children’s health situations and parents’ knowledge of vaccination (ID 1, 2), not to vaccination, as doctors vaccinate in a standard fashion (ID 7).

‘Coupling’ is a personal health problem in which a disease is incubated before vaccination and arises even without vaccination. Parents should be blamed, as they do not read the contraindications precisely, do not provide children’s health situations and do not follow guidelines. (ID 2)

These vaccines are safe. The qualifications of doctors, information records of vaccination, and equipment meet the standards. (ID 7)

The online medical experts proposed an inversion principle of the burden of proof, namely, that doctors should provide sufficient warrants to identify whether their actions amount to medical malpractice, as parents lack the expertise required to conceptualize risks and prove doctors wrong (ID 10, 11).

Doctors who are unable to provide logical evidences to substantiate their vaccination behaviours should be accountable for vaccine incidents. (ID 11)

Some parents argued that most children were diagnosed with coupling symptoms without persuasive evidence (ID 14). One self-defined victim accused coupling as being a tool used by experts to pass the buck, as her child had no allergic history or contraindications (ID 18).

There were seventeen vaccine cases all diagnosed as coupling in 2017, not related to vaccination, causing public distrust. (ID 14)

Clinicians recognised disputes over coupling diagnoses, but indicated difficulty in defining responsibility. According to some, the state should improve the compensation standards for vaccination-induced side effects (ID 5, 6, 9).

The complexity of personal physiques, the professionalism of vaccination and environmental vulnerability all make it difficult to allocate blame. The state should provide more financial relief instead of responsibility-defining. (ID 6)

Along similar lines, parents pointed to issues in the current compensation system and called for updating it to improve compensation amounts, simplifying the application procedures, and creating commercial insurance mechanisms as complements (ID 15, 16).

Terms for applying compensation are extremely harsh, and victims get less compensation [as a result]. Financial relief should be improved, including for coupling and general reaction, procedures [should be simplified], and commercial insurance [should be introduced]. (ID 16)

In summary, CDC experts tended to diagnose vaccine incidents as coupling symptoms unrelated to vaccination, while parents opposed coupling diagnoses as unpersuasive and argued that they were tools employed by doctors to pass the buck. The inversion of the evidentiary burden proposed by online medical experts is a possible solution, but considering the difficulty of responsibility-defining, clinicians and parents suggested a compensation system update instead.

## 4. Discussion

This qualitative study indicates that medical experts, journalists, and lay people have different perspectives on vaccination hesitance in China. Most interviewed CDC experts and clinicians, as well as two parents, considered that the strict regulation has enabled vaccines to become safe for the general public, despite a few flaws in some regional areas. They favored the National Immunisation Program (NIP) based on how it was logistically organized, including signed informed consent, single shots of vaccines, and the absence of antibody tests. They also justified past vaccine crisis responses as transparent and rational. The rest of the parents, the online medical experts, and all self-claimed vaccination victims presented different views. They considered vaccines to be unsafe and risky for their children, as vaccination doctors inappropriately kept vaccines in cold storage and some manufacturers were known to have produced invalid vaccines. They also criticized the compulsory signing of informed consent forms as a way to exempt doctors from responsibility for vaccination incidents. Contrary to the CDC experts and clinicians, these parents also preferred combined shots of vaccines and performing antibody tests on their children. They were not convinced by the responses of experts and public health bodies to vaccination incidents and called for more transparency to restrain the buck-passing of risk assessment experts and public engagement to attune to public benefits in crisis responses. Journalists and online medical experts mentioned the same risks in vaccine safety, vaccination organizations, and crisis responses as these parents, and they considered parental mistrust in the NIP to be a result of a failure of science popularization in China.

The analysis indicates that, while medical doctors and parents do not present themselves as homogeneous groups, the perspectives of experts and laypersons on vaccine hesitancy in China differ. To maintain a high vaccination rate, medical experts stress the safety of vaccines and the scientific organization of immunization, and legitimatize incident responses. Vaccination hesitance is ascribed to negative media reports about vaccination. In line with this, medical experts propose science popularization to mitigate parental vaccine hesitance. Medical experts articulate a ‘deficit-model’’ of communication, which considers expert knowledge as a standard and lay perspectives as irrational and lacking adequate knowledge [27,28]. The deficit model assumes that expert knowledge should be popularized for lay people, in order to stimulate rational choices. However, according to several scholars, this model reproduces instead of reduces public distrust in experts [29,30,31,32].

To mitigate public distrust in experts in China, in 2006, the term ‘science communication’ was introduced to replace ‘science popularization’, in order to emphasize the interaction between scientists and citizens in China, instead of top-down communication [33]. The massive science popularization campaigns that were embedded in political rhetoric were conducted to fight superstition, improve the low health literacy of Chinese citizens, and serve ‘the communist ideology’, and the notion of science communication aims to depart from that history. Nonetheless, according to Chen et al. [33], in practice, science communication in China remains a unidirectional transmission of knowledge from scientific experts to the public. China has put scientific expertise in an authoritative position since reform and the opening-up policy in China in the late 1970s, and governments invite ‘policy-compatible’ scientists as their spokespeople to ‘speak truth to power’ [34]. The close interaction between the state and science may put citizens at risk of harm [30,31]. One can also observe these characteristics of science communication with respect to vaccination hesitance. Symmetrical communication about vaccination incidents is difficult [35]. The Chinese state, out of fear of erosion of its authority, tends to prevent citizens from engaging in discussions on the vaccination program. State-allied experts are reluctant to get involved in open debates on the NIP, as they worry about a loss of credibility caused by societal skepticism [36].

Deficits in bilateral communication and public engagement about the NIP have stimulated the development of informal forums where experts and laypeople initiate discussions over vaccination hesitance on the Internet, such as the Sina Weibo, Zhihu, and Tianya communities, with less political restraint. According to Liang et al. [37], some public experts and journalists have discussed social justice issues in the NIP on these forums, such as adverse vaccination-induced events, potentially unsafe vaccines, and parent hesitance. Independent experts and journalists give voice to the laity, which expands the influence of independent experts and journalists. This informal practice is considered to be an instrument to compensate for deficiencies in public discourse in the formal system of the quasi-authoritarian state [38,39]. Our analysis shows that the construction of private, informal forums for discussing issues like vaccination implies that public engagement is not entirely absent in the largely authoritarian regime of China.

The coronavirus disease (COVID-19) pandemic in China might increase vaccine acceptance and mitigate vaccine hesitance among parents temporarily, as Severe Acute Respiratory Syndrome (SARS) did in 2003 [40,41]. A new study of vaccination hesitance in the context of COVID-19 is required to explore whether a similar diversity of perspectives is reported, whether the history of vaccination scandals is still resonating in the context of COVID-19, and whether there is more consensus about vaccination. Despite the changing context of vaccination issues, our study suggests a need for more open dialogues between experts and parents about their experiences with and insights into vaccination and this need may be even stronger in the context of COVID-19. Although the current Chinese political system, especially the close ties between the state and experts, makes such dialogues difficult, decision-makers and experts have realized the importance of more open communication about the public health crisis in mitigating public distrust during COVID-19 control in China. The initial response of Wuhan state to minimize information transparency when the first cases of COVID-19 were identified turned out to increase public distrust in public health, instead of increasing public trust, and this stimulated more openness [42]. Increasingly, medical experts have developed communication and interaction with the public about the management of COVID-19 via various channels: They have communicated on social media platforms and via media interviews and become actively involved in debates about controversial topics. Therefore, future studies should explore in depth how expert–lay communication about vaccination hesitancy in China develops in China in the context of COVID-19.

### Limitations of this Research

This is the first qualitative study to provide an in-depth understanding of stakeholder perspectives on vaccine hesitancy in China. The results of this qualitative study must be considered against the background of its limitations. First, this study aimed to explore the dynamics of vaccine hesitancy in China. For that purpose, we interviewed diverse stakeholders to understand their attitudes and perspectives on vaccines, vaccine safety, and the practice of vaccination. This generated varied and rich personal accounts. Compared with previous studies about vaccination hesitancy, our study not only points to concerns of stakeholders about unsafe vaccines, inappropriate medical conduct, and negative media reports, but also to concerns about regulatory deficits in professional conduct, unidirectional risk communication between experts and parents, limited information transparency following a crisis, and doctors passing the buck for vaccination-induced side effects. While it will be interesting to expand the sample size to acquire new perspectives on vaccine hesitancy in China in due course, it is important to realize that, due to COVID-19, the context of studying vaccination hesitancy has changed, and that new studies must consider these changes in the interpretation of results. Second, to understand multiple stakeholders’ perspectives on vaccine hesitance, we also included rich and detailed online vaccination stories of three parents. As these parents consider their children to be victims of vaccination, these stories are even more critical than others. Third, this study focused on examining vaccine hesitancy in urban areas. However, according to Wagner [43], the determinants of vaccine hesitance varied by region. Wagner demonstrated that parental vaccine hesitance was also rising in the rural areas of China, and the rates and determinants of vaccine hesitancy differed in the context of cities and rural areas [44,45]. To obtain insight into vaccine hesitancy in the various contexts of China, future studies should glean empirical evidence of vaccine hesitance in rural areas.

## 5. Conclusions

A large gap exists between medical expert and lay perspectives on vaccination. Vaccination experts align themselves with the political aim of raising vaccination rates through improvement of public scientific literacy (as such, they, alongside the Chinese government, ‘see like a state’ and focus on formal standardized metrics over reciprocal engagement [46]). In contrast, the public displays distrust towards experts, who are not sensitive to the doubts and criticisms of parents after severe vaccination incidents and simply repeat the state-endorsed view that the NIP is safe. Our analysis indicates that expert–lay relationships in China would benefit from less state influence on vaccination programs, as that would provide space for an open and sincere dialogue between experts and lay people that enables parents to express their concerns about vaccination programs and experts to respond to those concerns in a fair way.

## Figures and Tables

**Table 1:** Participant characteristics and descriptions of their function.

Interviewee ID	Gender	Description of Function
ID 1	Female	Expert in Shanghai CDC
ID 2	Female	Expert in Xinyang CDC
ID 3	Male	Clinician in Shanghai Tenth People’s Hospital
ID 4	Male	Clinician in Wuhan Union Hospital Vaccination Clinic
ID 5	Female	Clinician in Xinyang Central Hospital
ID 6	Female	Clinician at Children’s Health Clinic in Xinyang First People’s Hospital
ID 7	Female	Clinician in Shanghai Hongkou Community Healthcare Service
ID 8	Male	Clinician in Xinyang Community Healthcare Service
ID 9	Male	Clinician in Wuhan Biotechnology Co., Ltd.
ID 10	Male	Online medical expert
ID 11	Male	Online medical expert
ID 12	Male	Journalist in Wuhan Jingchu media
ID 13	Female	Journalist in Xinyang Economic Daily media
ID 14	Female	Mom with a 5-year-old daughter
ID 15	Male	50-year-old man, suffered from infantile paralysis in childhood
ID 16	Female	Mom with a 1-year-old son
ID 17	Male	Father with two children

**Table 2:** Characteristics of online informants and descriptions of their function.

Informant ID	Gender	Function	UCL
ID 18	Male	Self-defined victim: Parent with a child who suffered from disability after Polio vaccination	http://bbs.tianya.cn/post-free-4708430-1.shtml
ID 19	Female	Self-defined victim: Parent with a daughter who suffered epilepsy after rabies vaccination	http://bbs.tianya.cn/post-develop-2334312-1.shtml
ID 20	Female	Self-defined victim: Parent with a baby who suffered infantile spasms after DPT vaccination	https://www.weibo.com/ttarticle/p/show?id=2309404181411853683814
